# Survival Advantage Following TAG-72 Antigen-Directed Cancer Surgery in Patients With Colorectal Carcinoma: Proposed Mechanisms of Action

**DOI:** 10.3389/fonc.2021.731350

**Published:** 2021-12-07

**Authors:** Charles L. Hitchcock, Stephen P. Povoski, Cathy M. Mojzisik, Edward W. Martin

**Affiliations:** ^1^ Department of Pathology, College of Medicine, The Ohio State University, Columbus, OH, United States; ^2^ Division of Surgical Oncology, Department of Surgery, College of Medicine, The Ohio State University, Columbus, OH, United States

**Keywords:** colorectal carcinoma, TAG-72, surgery, survival, glycosylation, immunosuppression

## Abstract

Patients with colorectal carcinoma (CRC) continue to have variable clinical outcomes despite undergoing the same surgical procedure with curative intent and having the same pathologic and clinical stage. This problem suggests the need for better techniques to assess the extent of disease during surgery. We began to address this problem 35 years ago by injecting patients with either primary or recurrent CRC with ^125^I-labeled murine monoclonal antibodies against the tumor-associated glycoprotein-72 (TAG-72) and using a handheld gamma-detecting probe (HGDP) for intraoperative detection and removal of radioactive, i.e., TAG-72-positive, tissue. Data from these studies demonstrated a significant difference in overall survival data (p < 0.005 or better) when no TAG-72-positive tissue remained compared to when TAG-72-positive tissue remained at the completion of surgery. Recent publications indicate that aberrant glycosylation of mucins and their critical role in suppressing tumor-associated immune response help to explain the cellular mechanisms underlying our results. We propose that monoclonal antibodies to TAG-72 recognize and bind to antigenic epitopes on mucins that suppress the tumor-associated immune response in both the tumor and tumor-draining lymph nodes. Complete surgical removal of all TAG-72-positive tissue serves to reverse the escape phase of immunoediting, allowing a resetting of this response that leads to improved overall survival of the patients with either primary or recurrent CRC. Thus, the status of TAG-72 positivity after resection has a significant impact on patient survival.

## Introduction

On a global basis, colorectal carcinoma (CRC) is ranked third in incidence and second in cancer-related mortality (1). Of the non-keratinocytic tumors, in the United States, CRC ranks fourth in overall incidence and second in mortality (2). Approximately 80% of newly diagnosed patients undergo surgical resection with “curative” intent. However, the patient outcome varies despite having the same surgical procedure performed by the same surgeon and having a tumor with the same morphologic features and pathologic stage (3). The incidence of recurrent CRC varies from 20% to 50% of cases following surgery with curative intent, the majority of which occur within the first 3 years following surgery (4–6). Curative resection of cancer requires the removal of “all” tumor-involved tissues. Differences in patient outcomes can arise from a lack of accurate assessment of the extent of disease by preoperative imaging (7), surgical exploration (8), pathologic staging (9), and tumor biology (10). Together, these variabilities result in an inaccurate evaluation of the individual patient’s prognosis. This inaccuracy is not a new problem, as evidenced by over 60 years of findings from second-look surgeries for recurrent disease in otherwise asymptomatic patients (11, 12).

## Antigen-Directed Cancer Surgery

In the late 1980s and 1990s, a series of clinical trials was undertaken with the goal to improve the surgeon’s ability to intraoperatively detect and remove tumor-involved tissues from patients with either recurrent or primary CRC (13, 14). The studies combined the use of ^125^I-labeled murine monoclonal antibodies (mMoAbs) against the tumor-associated glycoprotein-72 (TAG-72) antigen and a handheld gamma-detecting probe (HGDP) for intraoperative detection of TAG-72-positive tissue. We refer to these studies as TAG-72 Antigen-Directed Cancer Surgery (ADCS) rather than the previously described Radioimmunoguided Surgery (RIGS) (15, 16).

In brief, the protocols included blocking the thyroid gland uptake of ^125^I preoperatively. Early studies called for patients to be injected intravenously with ^125^I-anti-TAG-72 mMoAb B72.3, whereas patients in later studies received ^125^I-anti-TAG-72 mMoAbs CC49 or CC83 (15, 16). Serial precordial counts using the HGDP ensured that an optimal tumor-to-background ratio occurred before taking the patient to surgery within 28 days after injection. The surgeon first used traditional exploration techniques (i.e., inspection and palpation) to explore the abdomen and pelvis and then declare the findings and surgical plan. The surgeon then used the HGDP to resurvey the pelvis and abdomen, which often led to the detection of occult (residual) tumors and a change in the surgical plan.

## TAG-72 Protein

TAG-72 was originally isolated from xenografts of the human colon carcinoma cell line LS-174T through binding of a mMoAb called B72.3 (17–19). The B72.3-binding isolate was characterized as a high-molecular weight (>1,000,000 kD) protein with extensive glycosylation consistent with a mucin. Further work identified the TAG-72 epitopes recognized by several of these mMoAbs as different O-linked glycan antigenic structures (17, 18). B72.3 binds ovine submaxillary mucin, a glycoprotein rich in the sialyl-Tn (NeuAcα2-6GalNAcα1-Ser/Thr, STn) tumor-associated carbohydrate antigen (TACA). This binding was eliminated by sialidase treatment and inhibited by both STn and the GalNAcα1-Ser/Thr (Tn) precursor structure. In contrast, mMoAbs to the Thomsen-Friedenreich (TF) antigen (Galβ1-3GalNAcα-O-Serine/Threonine) did not inhibit B72.3 binding (20). Additional work by Reddish et al. (21) showed that STn-O-serine dimeric and trimeric clusters were recognized by mMoAb B72.3 better than the monomeric structure. The finding of cross reactivity with dimeric Tn-O-serine is consistent with earlier findings that B72.3 agglutinated red blood cells (22).

The discovery of second-generation mMoAbs to B72.3-purified TAG-72 demonstrated a series of overlapping but unique glycan antigenic epitopes (23–25). We subsequently used this second-generation mMoAb, called CC49, in our ADCS studies. The glycan antigenic epitope recognized by CC49 overlaps with that recognized by the B72.3, as it had a strong reactivity with dimeric STn-O-serine/threonine and less reactivity with Tn-O-serine dimers; it also lacked reactivity to monomeric forms of STn-O-serine or Tn-O-serine (21). In addition, several studies demonstrated that CC49 has a higher binding affinity for the core-1 sialylated glycan NeuAcα2-3Galb1-3GalNAc (sTF) than STn (26–29). The fact that both mMoAbs bind equally to periodate-treated mucins indicates that the overlapping epitopes do not include the sialic acid glycerol side chain in its free or O8′ or 09′ acetylated forms (29).

**Figure 1:**
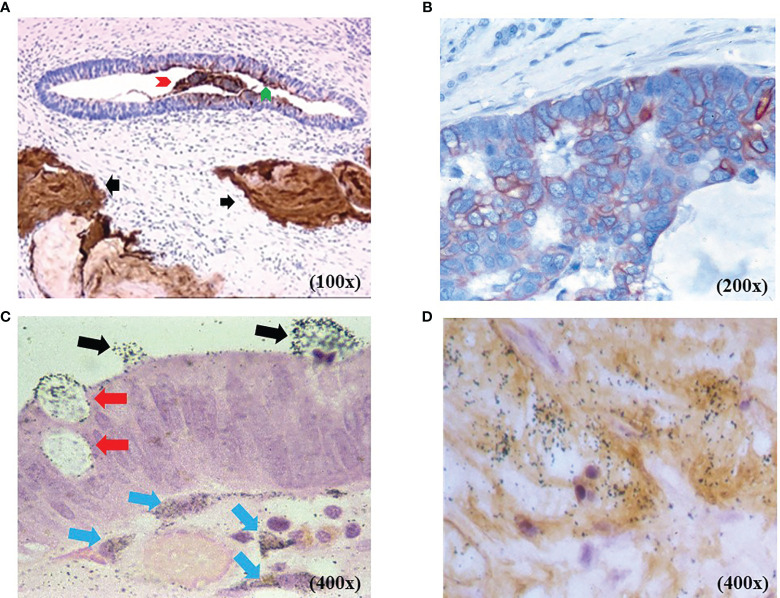
TAG-72 expression in colorectal adenocarcinoma [colorectal carcinoma (CRC)]. **(A)** Non-mucinous CRC with murine monoclonal antibody (mMoAb) CC49 immunohistochemical (IHC) staining (brown) demonstrates TAG-72 in malignant glands and in mucin lakes in the tumor microenvironment (TME) (black arrows). The malignant gland exhibits TAG-72 expression in the luminal contents (red arrow head), cytoplasmic vacuoles, and the tumor cells’ luminal surface (green arrow head). **(B)** Non-mucinous CRC with mMoAb CC49 IHC staining (brown) demonstrates TAG-72 outlining the plasma membrane of the malignant cells. **(C)** Black autoradiography silver grains (ARGs) of the injected mMoAb ^125^I-CC49 demonstrates ^125^I-CC49-bound TAG-72 in cytoplasmic secretory vesicles (red arrows) in CRC cells as they move from the cytoplasm to the lumen of a malignant gland (black arrows). Below the malignant cells are ARGs in the cytoplasm of dendritic-shaped cells (blue arrows) in TME. **(D)** Co-localization of the injected ^125^I-mMoAb CC49 (black ARGs) overlapping with the IHC staining (brown) for TAG-72 demonstrates the TAG-72 antigenic epitope in the TME of a CRC.

Immunohistochemical (IHC) staining with either mMoAb B72.3 or CC49 demonstrated TAG-72 expression in various carcinomas and only in normal secretory endometrium (30–32). IHC staining and autoradiography (**Figure 1**) show TAG-72 in the cytoplasm of CRC tumor cells, in their luminal secretions, and in the tumor microenvironment (TME). IHC staining demonstrates TAG-72 dispersed in the plasma membranes of apical, lateral, and basal surfaces of tumor cell luminal debris (**Figures 1A, B**). In addition, TAG-72 occurs in the luminal debris of malignant glands (**Figure 1A**). TAG-72-positive secretions are evidenced by positive IHC staining TAG-72 of mucin lakes in the TME of non-mucinous and mucinous carcinomas, as well as in cytoplasmic vacuoles, and luminal CC49 confirms the IHC staining results with the respective mMoAb (**Figure 1C**) (33). **Figure 1C** depicts the black silver autoradiography grains (ARGs) representing the injected ^125^I-labeled mMoAb CC49 binding to TAG-72 in secretory vesicles that are migrating to the apical surface and released into the lumen of a malignant gland. In addition, ARGs occur in cells and the extracellular matrix of the TME along the basal surface of the tumor cells. **Figure 1D** demonstrates the co-localization of ARGs and IHC staining of TAG-72 in the TME and cells.

**Figure 2:**
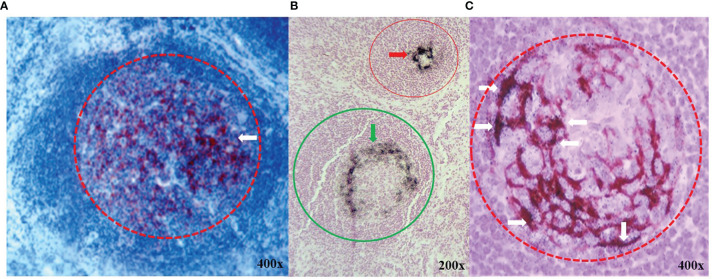
TAG-72 expression in germinal centers of lymphoid follicles in extraregional tumor draining lymph nodes. **(A)** Immunohistochemical (IHC) staining with murine monoclonal antibody (mMoAb) CC49 (dark red) demonstrates early polarization (white arrow) of TAG-72 toward the periphery of a germinal center (red circle) in a reactive lymphoid follicle. **(B)** Black autoradiography silver grains (ARGs) located in demonstrate the injected ^125^I- mMoAb CC49 in two germinal centers (red and green circles). TAG-72 distribution varies from an acentric pattern (green arrow) in the germinal center of a reactive lymphoid follicle (green circle) as compared to a dense circular peripheral pattern in the germinal center (red arrow) of a smaller lymphoid follicle (red circle). **(C)** mMoAb CC49 IHC staining (brown) and black ARGs demonstrate co-localization of TAG-72 (white arrows) in an acentric dendritic pattern in a reactive germinal center (red dashed circle).

TAG-72 expression in tumor-draining lymph nodes (TDLNs) is similar to that of the tumor itself (**Figure 2**). Reactive germinal centers (**Figures 2A** **–C**), sinus histiocytosis, and paracortical hyperplasia (not shown) are prominent histologic features in regional and extraregional TDLNs in CRC. In the case of CRC, TAG-72 localizes to the germinal centers of the TDLNs. IHC staining and autoradiography commonly demonstrate TAG-72 in a dendritic or eccentric distribution within the germinal centers (**Figures 2A** **–C**). IHC staining with mMoAbs B72.3 or CC49 demonstrates that TAG-72 expression is similar to that of other pan-carcinoma antigens in adenocarcinomas (34). In addition, it is important to note that not all CRC tumors take up anti-TAG-72 mMoAbs. The mMoAb B72.3 and mMoAb CC49 localized in 75% and 86% of patients with primary CRC, respectively. In contrast, mMoAb B72.3 and MoAb CC49 localized in 63% and 97% of patients with recurrent CRC (14).

## Patient Survival

**Figure 3:**
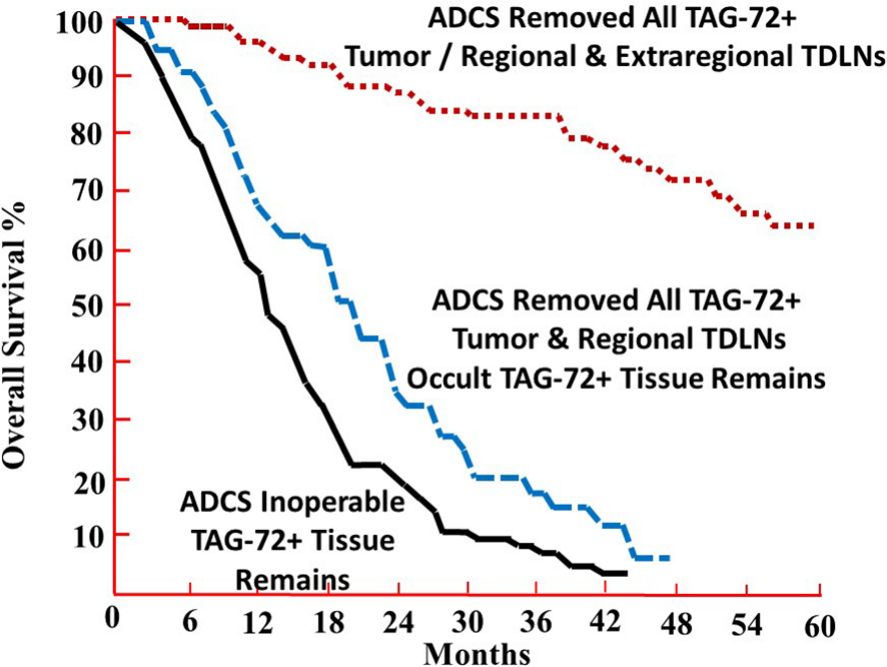
The 5-year overall survival (OS) percentage of patients undergoing TAG-72 antigen-directed cancer surgery. The 5-year OS of 212 patients with recurrent or primary colorectal carcinoma was injected with murine monoclonal antibody (mMoAb) ^125^I-B72.3 or ^125^I-CC49 (37). Complete removal of all TAG-72-positive tissue provides a highly significant 5-year OS advantage when compared to those patients with retention of grossly evident tumor and TAG-72-positive tissue (p < 0.0001) (black line) or occult TAG-72-positive tissue (no residual grossly evident tumor) (p < 0.0025) (blue dashed line) upon completion of surgery.

The overall survival (OS) data provide direct evidence that using radiolabeled anti-TAG-72 mMoAbs to intraoperatively determine the extent of disease had a significant clinical impact with the removal of all the TAG-72-positive tissue (35–40). **Figure 3** depicts the 5-year OS of 212 patients with primary or recurrent CRC intravenously injected with mMoAbs ^125^I-B72.3 or ^125^I-CC49 for TAG-72 ADCS (37). The patients were divided into three groups based on the HGDP evidence of tumor at the end of surgery. The largest group of 95 (44.8%) patients had grossly evident tumor and TAG-72-positive tissue remaining upon completing the surgical procedure (black line). Carcinomatosis and unresectable liver or lung metastases were common among the patients with primary CRC in this group. Patients with recurrent CRC in this group who had unresectable pelvic, abdominal, and thoracic metastases did not tend to survive beyond 3 years. In addition, this study demonstrated that mMoAb ^125^I-B72.3 detected the unresectable disease in patients with recurrent CRC that would have undergone unwarranted surgery based solely on traditional detection techniques (36).

In stark contrast are the 74 (34.9%) patients with primary or recurrent CRC who lacked evidence of gross tumor and TAG-72-positive tissue at the end of surgery (red dotted line). For those patients with primary CRC, the 5-year OS was independent of the pathologic stage (38). For patients with recurrent disease, the 5-year survival identified a population of patients who benefitted from TAG-72 ADCS (13, 14).

**Figure 3** also identifies a group of 43 (20.3%) patients lacking grossly evident tumor while retaining TAG-72-positive tissue, consistent with unresected lymph nodes, at the end of surgery (blue dashed line). A follow-up study identified the recurrent disease in previously unresected, TAG-72-positive, extraregional TDLNs (41). These results demonstrate the clinical implications of the relationship between TAG-72-positive extraregional TDLNs and the extent of disease in CRC patients. Overall, these results indicate that TAG-72 ADCS detects and localizes occult diseased tissue left behind when only using more traditional surgical techniques (e.g., inspection and palpation).

**Figure 4:**
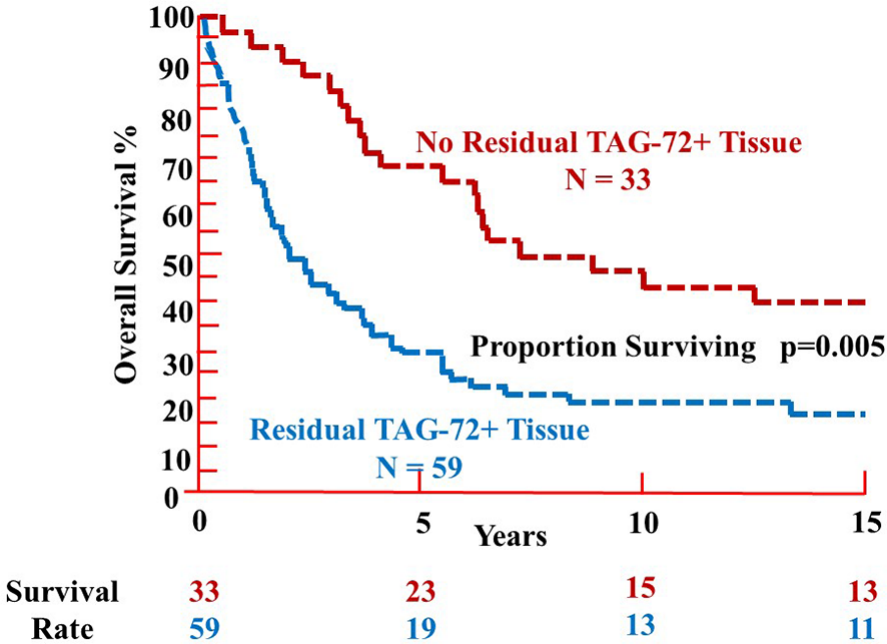
Overall survival of patients with colorectal carcinoma following TAG-72 antigen-directed cancer surgery. Ninety-two primary colorectal carcinoma patients were injected with either murine monoclonal antibody (mMoAb) ^125^I-CC49 or mMoAb ^125^I-CC83 (16). There was a significant difference (p = 0.005) in the proportion surviving between those patients with no residual TAG-72-positive tissue at the end of surgery (red dashed line) as compared to those patients where residual TAG-72-positive tissue (blue dashed line) remained at the end of surgery. The survival rate at 5 years was 70% for those patients without residual tumor (upper red values) as compared to 32% for patients with residual TAG-positive tissue (lower blue values). However, there is no significant difference in the survival rate after 10 and 15 years of follow-up.

Analysis of 92 patients with primary CRC injected with mMoAb ^125^I-CC49 demonstrated a significant difference in the % OS relative to the presence or absence of residual TAG-72-positive tissue beyond 5 years, at 10 years (p = 0.002) and 15 years (p = 0.003) (**Figure 4**) (16). Analysis of OS *vs*. pathologic stage (pStage) required grouping patients relative to the absence of metastatic disease (pStages 0, I, and II) or presence of metastatic disease (pStages III and IV). As expected, patients with visceral metastases (pStage IV) were significantly (p = 0.03) more prevalent among those patients with residual TAG-72-positive tissue at the end of surgery (16).

## Antigen-Directed Cancer Surgery and Extent of Disease

Does TAG-72-positive tissue equate with the presence of shed antigen? The answer to this is yes, and we consider it a significant component of the extent of disease. TAG-72 circulating in patients with adenocarcinomas, including CRC, is demonstrated by the elevated levels of TAG-72 glycoprotein in the serum and effusions (42, 43) and by IHC staining demonstrating TAG-72 in tumor lymphatic vessels (44). TAG-72 in TDLNs co-localizes mMoAb ^125^I-CC49 in germinal centers with a crescentic dendritic pattern, suggestive of a T-helper 2 (Th2) immune response or a central distribution (**Figure 2**) rather than the Th1 tumor-associated immune response (TAIR) (11). Together, the TAG-72 ADCS results (reviewed in 13, 14) and the morphologic data support our hypothesis that TAG-72 positivity in the tissue equates with the extent of disease of CRC.

Is TAG-72 ADCS a better predictor of extent of disease than routine pathologic staging based on conventional surgery? Data from our ADCS cases point out that the extent of disease extends beyond what surgeons consider as the normal area of resection. Tissue involved with tumor often goes undetected by preoperative imaging, by the surgeon’s visual inspection or manual palpation, and/or by a lack of exploration. In 41 primary CRC cases, the surgeon alone identified 45 sites as compared to 153 sites detected using the HGDP to identify mMoAb ^125^I-CC49 in the tissue (45). In a similar comparison in 45 cases of recurrent CRC, conventional methods identified 116 sites as compared to 184 sites using the HGDP to identify mMoAb ^125^I-CC49 in the tissue. The percentage of TAG-72-positive lymph nodes from the area of the gastrohepatic ligament (66%) and celiac axis (~50%) was similar for both primary and recurrent cases. The ratio of tumor-involved liver to these three lymph node-bearing locations was 1:3 and 1:1 in primary and recurrent cases, respectively. Several non-ADCS studies (reviewed by 46) identified metastatic CRC in similar lymph node groups, as noted above, and that removal of these nodes improved patient outcomes. However, recurrent disease occurred in these areas when TAG-72-positive tissue was purported to be negative on frozen sections that remained behind at the end of surgery (41). Similarly, non-ADCS data demonstrated that lymph node recurrences outnumber those in the liver and lungs and occur alone or in combination with distant metastases in over 90% of the 835 cases that developed recurrent CRC out of the 4,023 patients who underwent curative surgery (5).

The ADCS results beg the question: Does TAG-72-positive tissue equate with the presence of metastatic tumor cells? The answer is controversial. Many TAG-72-positive TDLNs lacked tumor cells based on routine pathologic studies, indicating that the answer to this question is no (46, 47). Our results from an unpublished survey of 599 consecutive TAG-72-positive specimens from 92 patients with either primary or recurrent CRC suggest that the answer is no. In non-lymphoid tumor deposits, tumor cells were present in 92.5% (136/147) of hematoxylin and eosin (H&E)-stained sections submitted for routine pathologic examination. In contrast, only 15.7% (71/452) of TAG-72-positive lymph nodes contained tumor cells. This difference was highly significant (p < 0.00001). These results appear to support the impression that the lack of tumor cells in TAG-72-positive lymph nodes submitted for routine histopathology was indicative of false positivity (44, 46–48). However, numerous variables account for “false-negative” results, including lymph node size, size and location of the metastasis in the node, and the number of sections taken (9, 49–52).

Secondly, routine tissue processing, sectioning, and H&E staining induce errors in sampling and sensitivity. Thirdly, H&E-negative TAG-72-positive lymph nodes may contain unrecognized tumor cells because a routine single 4–5-mm section evaluated by the pathologist represents less than 0.1% of a 1-cm-diameter node (49). Cytokeratin IHC staining studies, with or without step sectioning, detected tumor cells in otherwise H&E-negative lymph nodes (49, 51, 52). However, even these studies suffer from errors in sampling and sensitivity (49). More sensitive flow cytometry, cell sorting, and RT-PCR studies have identified tumor cells’ presence in otherwise “H&E-negative” TAG-72-positive lymph nodes (37, 53–55).

## Mechanisms Underlying Survival Results From TAG-72 Antigen-Directed Cancer Surgery for Colorectal Carcinoma

### Aberrant O-Linked Glycosylation of Mucins in Colorectal Carcinoma

**Figure 5:**
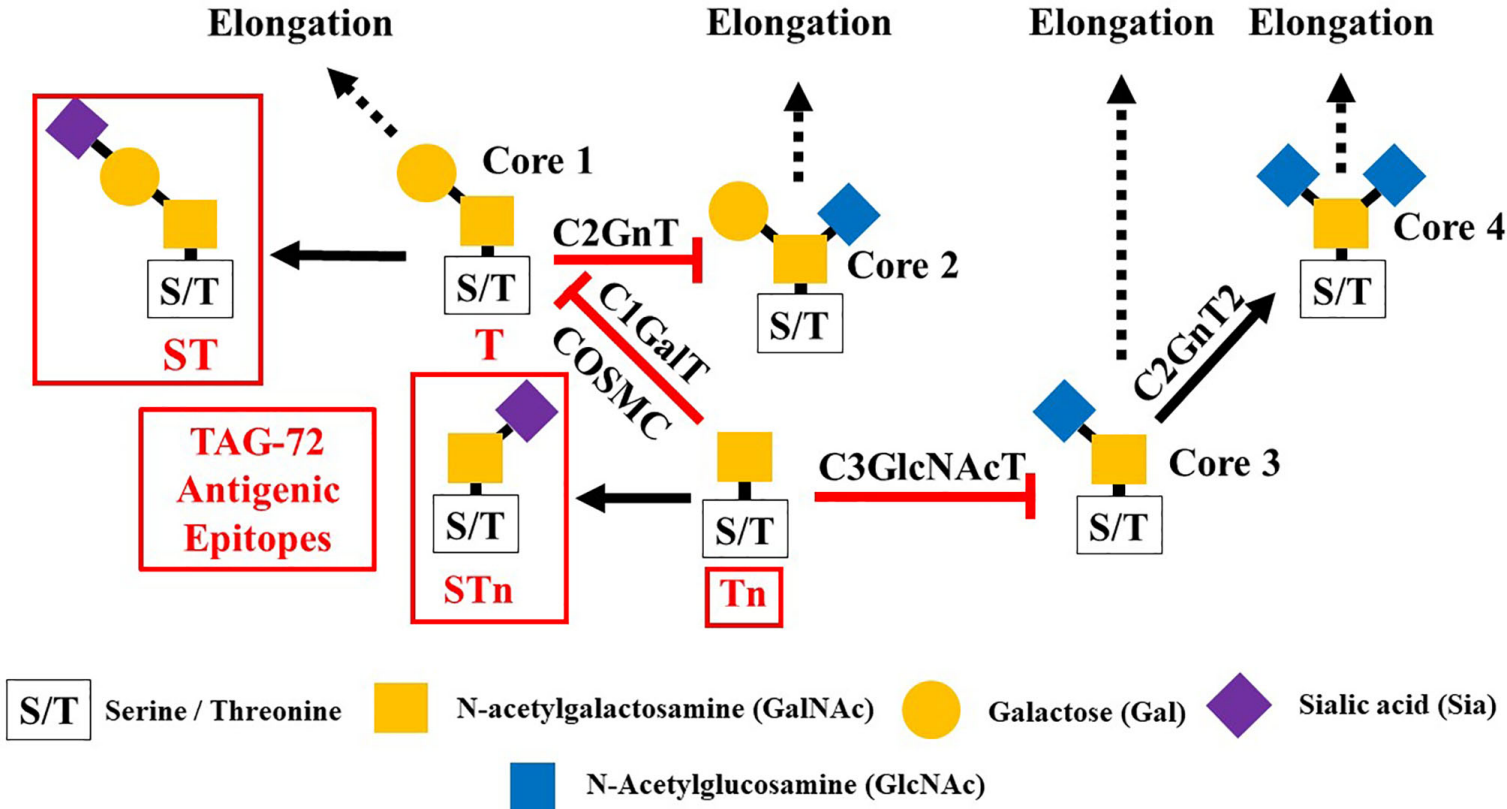
Formation of Core structures in O-linked glycosylation of mucins. Normal O-linked glycosylation begins with the addition N-acetylgalactosamine (GalNAc) to a serine or threonine (S/T) amino acid on core protein. Core 1 Gal-transferase (C1GalT), with the help of the COSMC chaperone protein, adds Gal to the GalNAc-S/T to form the Core 1, which in turn gives rise to Core 2 with the addition of GlcNAc to GalNAc that is catalyzed by Core 2 N-acetylglucosamine transferase (C2GnT). Core 3 N-acetylglucosamine transferase (C3GnT) is the only enzyme that catalyzes the addition of GlcNAc to GalNAc to form Core 3. Core 4 is formed by the addition of another GlcNAc to the GalNAc of Core 3. The subsequent addition of sugars to these Cores [i.e., elongation (dash black arrows)] gives rise to the variable O-glycosylation pattern of mucins. The expression of Tn, STn, T, and ST glycans (red boxes) and loss of Core 3 are often the result of mutations of the transferase genes (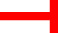) associated with the formation of Cores 1, 2, and 3, and the loss of C2GnT activity is also associated with hypermethylation of the gene for the chaperone protein SMC. The loss of function of one or more of these enzymes accounts for the presence of TAG-72 antigenic epitopes (red boxes) present in various carcinomas.

Glycosylation is the predominant posttranslation modification of cellular proteins and lipids. The complex process of O-linked-glycosylation of mucins begins with N-acetylgalactosamine (GalNAc) that is O-linked to either serine (S) or threonine (T) amino acids on the peptide that serves as the starting point for addition of sugars to form the precursors of the first four of the eight core structures (**Figure 5**) out of the possible. Aberrant O-glycosylation is a hallmark of carcinomas (56). This process gives rise to immunogenic glycans that are collectively refereed as tumor-associated carbohydrate antigens (TACAs) (57, 58). Mucins and other glycoproteins concurrently express TACAs, such as the truncated core Tn, STn, T, and ST, as well as Lewis (Le) blood group antigens Le^a/x^ and Le^b/y^ and their sialylated glycoforms (59). A given glycoprotein can express multiple different TACAs (60, 61). The accumulation of Tn, STn, T, and ST (**Figure 5**) is a characteristic feature of CRC and other carcinomas (62). The Tn, ST, and STn containing antigenic epitopes recognized by mMoAbs to TAG-72 occur early in the adenoma–carcinoma sequence and tumor progression (63–66). The accumulation of Tn and STn is associated with a mutation and with hypermethlyation of the *Cosmc* gene whose product is the chaperone protein Core 1 β3GalT-specific molecular chaperone (Cosmc). Cosmc is required for the proper function of the Core 1Gal-Transferase enzyme (C1GalT) to synthesize the T glycan (**Figure 5**) (67). In addition, Tn and STn accumulate with the mutation of the gene for N-acetylglucosamine transferase (C3GnT), which is the only enzyme generating the core 3 precursor glycan (**Figure 5**), and its loss is common in CRC (68). Radhakrishnan et al. (69) observed that hypermethlyation of the promoter of *Cosmc* is the “most prevalent cause” of Tn and STn formation in pancreatic cancer samples. They used an immortalized keratinocytic cell model to examine the phenotype roles of these truncated glycans. Their results demonstrated that truncated O-linked glycans alter cell adhesion and the RAS signaling pathways leading to cell proliferation, tissue invasiveness, and decreased apoptosis. Differences in the expression pattern may well be due to the presence or absence of such mutations.

IHC staining demonstrates that Tn and STn accumulate in over 85% of colorectal, pancreatic, and ovarian carcinomas and over 50% of the carcinomas of the lungs, cervix, esophagus, stomach, and breasts (23, 57, 70–72). Although aberrant glycosylation varies with the tumor type, the expression of the truncated glycans, Tn and T and their sialylated counterparts, is associated with a poor prognosis for patients with a carcinoma including CRC (57, 64–66, 72). This correlates with the observation that aberrant glycosylation, including Tn and STn, suppresses the TAIR (63, 64).

### Tumor-Associated Immune Response

The microenvironments of both the tumor and the TDLNs are a complex and intricate network of neoplastic, stromal, and infiltrating immune cells and their products. Through the interaction of these components, tumor cells progress to acquire the potency to evade ongoing immune responses by reducing immune recognition, increasing their resistance against immune attack, and creating an immunosuppressive tumor microenvironment.

The concept of tumor immunoediting helps explain the role of immune cells, especially dendritic cells (DCs), in tumor progression (73, 74). Elimination, or immunosurveillance, the first of the three “E” processes, equates with an effective TAIR that eliminates neoplastic cells. Here, the mechanism of the TAIR “outweighs” those of immunosuppression. The Equilibrium phase represents a balance between the mechanisms of elimination and immunosuppression that can last your years. Escape is the third phase of the immunoediting process and equates with clinically evident disease and suppression of the TAIR.

**Figure 6:**
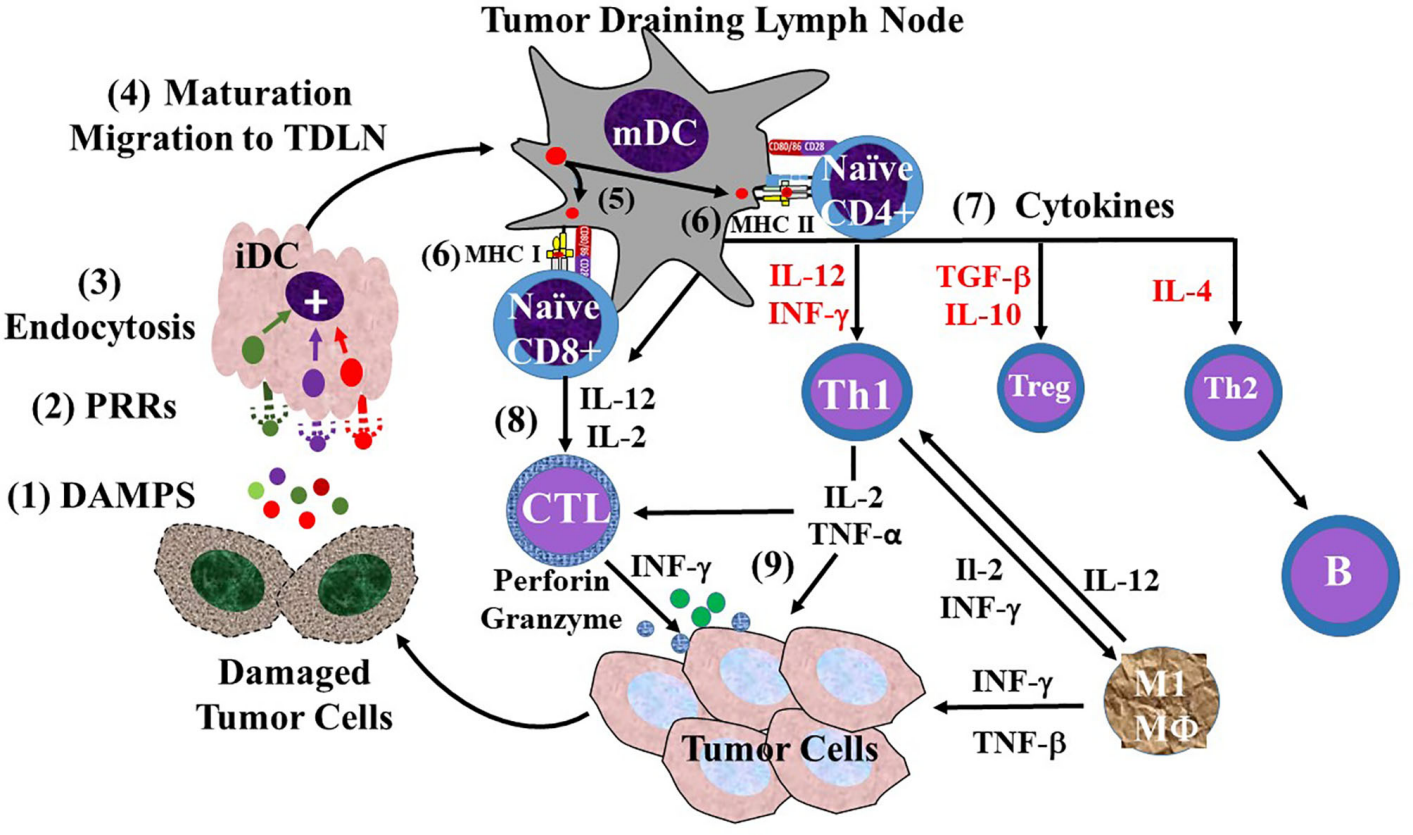
Overview of the sequence of events involving dendritic cells in the tumor-associated immune response (TAIR). The TAIR begins with natural killer (NK) cell and macrophage (not shown) inducing damage of tumor cells that (1) releases damage-associated molecular patterns (DAMPs) into the tumor microenvironment (2). The DAMPs bind to various types of pattern recognition receptors (PRRs) on immature dendritic cells (iDCs) (3), where their endocytosis leads to activation of iDC maturation (4). iDC maturation leads to morphologic changes, decreased endocytic activity, migration lymphatics to a tumor draining lymph node (TDLN). Once in the TDLN, the now mature dendritic cell (mDC) provides the three signals needed for activation of naive T cells. mDCs are capable of cross-presenting antigens (5) to both major histocompatibility complex (MHC I and MHC II) molecules of the surface. Signal 1 (6) is generated by the MHC molecule presentation of the antigen to the T-cell receptor (TCR) on naive CD 4 or CD8 T cells. Signal 2 is generated by the binding of co-stimulatory molecules CD80/86 on the mDC to CD28 on the naive T cells (7). Signal 3 is provided o the release of pro-inflammatory cytokines (e.g., IL-2, IL-12, IFN-γ) that activate naive CD4+ T-helper (Th) cells to differentiate into different populations based on the cytokine milieu (red text). These include IL-2 and TGF-β stimulated CD4 regulatory T cells (Treg), and IL-4 stimulated Th2 cells. However, IL-12 induced differentiation of naive CD4 cells into Th1 cells in the major antitumor effector T cells (8). Activation of naive CD8 by IL-12 and IL-2 leads to their differentiation into CD8+ cytotoxic T lymphocytes (CTLs). The Th1s and CTLs migrate back to the tumor cells *via* blood vessels (9). The tumor-associated immune response (TAIR) involves the Th1 cells activating M1 macrophages (MΦ), CTL release of perforin and granzyme, and release of IL-2 and TNF-α that work together to damage tumor beyond repair.

The TAIR is dependent on the ability of immature dendritic cells (iDCs) to become mature dendritic cells (mDCs) that migrate to TDLNs (**Figure 6**). In the TDLN, DC maturation gives rise to the three signals needed to activate naive CD4+ and CD8+ T cells to become effector T cells needed to combat tumorigenesis. We propose that binding of TACA to iDCs in the TME and TDLN leads to CRC progression and that the ability of TAG-72 ADCS to reverse this suppression of the TAIR accounts for the mechanisms that underlie our results.

### Dendritic Cells and the Tumor-Associated Immune Response

DCs aided by natural killer (NK) cells and macrophages (75) play a central role in bridging the innate and adaptive immune responses. Bone marrow precursors give rise to a heterogeneous group of DC subsets that vary in their developmental pathway, location, and phenotype (76). The various subpopulations of DCs are grouped as conventional (cDC1 and cDC2) and plasmacytoid (pDC). These three subsets differ in their expression of pattern recognition receptors (PRRs), surface markers, cytokine expression, and promotion of Th1 *vs*. Th2 *vs*. Th17 immune responses (77). These differentiated DC subsets exist functionally as either immature or mature. Subsets of iDCs are present in all tissues where they sample their environment for non-self molecules, pathogen-associated molecular patterns (PAMPs), and damage-associated molecular patterns (DAMPs) released by damaged or dead cells (78), as well as self-associated molecular patterns (SAMPs) (79) that bind to PRRs on iDCs and other antigen-presenting cells. iDCs primarily take up the bound molecular patterns by receptor-mediated endocytosis. This binding activates iDC maturation that leads to morphologic and phenotype changes and migration to lymph nodes.

### Normal Tumor-Associated Immune Response

In brief (**Figure 6**), the TAIR, as the first phase of immunoediting, eliminates neoplastic cells. It begins with tumor-associated antigens binding to PRRs on resident NK cell and M1-type macrophages (74). This binding induces tumor cell apoptosis and lysis that releases DAMPs into the TME. DAMPs bind to various PRRs that include carbohydrate-binding lectins and Toll-like receptors on iDCs and macrophages. The resulting activation of iDCs, primarily cDC1s (80), leads to a cascade of events leading to a pro-inflammatory TAIR (81). Activation of iDCs begins with phagocytosis or receptor-mediated endocytosis resulting in DC maturation and migration to TDLNs (82). Balan and Bhardwaj (83) postulated that, once in the TDLN, the now mDCs may transfer antigen-containing vesicles to resident cDC1 and cDC2 cells. The migrated mDCs and the resident DC subpopulations provide naive T cells with the three signals needed for their activation. Signal 1 is the cross-presentation of antigens bound to both major histocompatibility complex (MHC) I and MHC II molecules. MHC I-bound antigens are presented to naive CD8+ T cells by resident DCs and cDC1s (80, 84), while resident counterparts of cDC2s and cDC1s present antigen *via* MHC II molecules to naive CD4+ T cells (75, 85, 86). Signal 2 is generated by the binding of the CD80/86 co-stimulator molecules on mDCs to CD28 co-stimulator molecules of naive T cells. Signal 3 is provided by the release of various pro-inflammatory cytokines, the type of which is dependent on the antigen presented by the mDC. These cytokines include interleukin 12 (IL-12), primarily by cDC1s, as well as interferon gamma (IFN-γ), transforming growth factor beta (TGF-β), IL-4, and IL-10 that activate naive CD4+ Th cells to differentiate into different populations of effector CD4 Th cells based on the cytokine milieu (73, 86). IL-2 and IL-12 provide Signal 3 for activation of CD8+ cells. The effector T cells—CD8+ cytotoxic T lymphocytes (CTLs) and CD4+ T cells—in a Th1 response migrate back to the nascent tumor TME. In the TME, the CD8+ cytotoxic T cells release IFN-γ as well as perforin and granzyme that lyse the tumor cells. Th1 cells release pro-inflammatory cytokines support CTL and M1 macrophages provide cytokines for CTLs and M1 macrophage functions. Together, these three cells, among other cells in the TME, damage tumor cells, releasing DAMPs that start the process over again (86, 87).

### Tumor-Associated Immune Response Immunosuppression in Colorectal Carcinoma

We propose that the interaction of TACAs with innate immune cells plays a central role in the escape phase of the immunoediting characterized by the suppression of the TAIR. The tumor-associated glyco-code correlates with the heterogeneous expression patterns of Tn, STn, and ST and other TACA ligands on glycoproteins and glycolipids of cancer cells and their association with patient prognosis and binding to carbohydrate-binding receptors (i.e., lectins) on the cells of the innate and adaptive immune responses (**Table 1**) (60, 88). Our hypothesis centers on the impact this binding has on the CRC-related TAIR.

**Table 1:** Colorectal carcinoma (CRC) tumor-associated carbohydrate antigens (ligands) and the lectin-binding cells Tumor-Associated Carbohydrate Antigens (TACA), Serine/threonine (S/T), N-acetyl-galactosamine (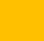), Sialic Acid (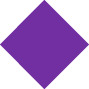), Galactose (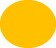), Fucose (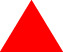), N acetyl-glucosamine (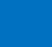), Radicals – (i.e., carbohydrates) closer to the serine/threonine (R), Macrophage Galactose-Type Lectin (MGL), Sialic Acid Recognizing Ig-like Lectins (Siglecs), Dendritic Cell-Specific ICAM-3 Grabbing Non-Integrin (DC-SIGN), Macrophage (MΦ), Immature Dendritic Cell (iDC), Monocyte (Mono), Natural Killer (NK) Cell.

TACA	Structure	Lectins	Lectin-Carrying Cells
Tn	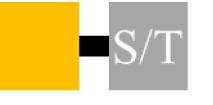	**MGL**	**iDC, MΦ**
STn	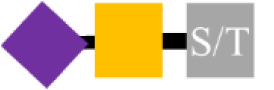	**Siglecs** **MGL**	**iDC, MΦ, Mono, NK**
ST	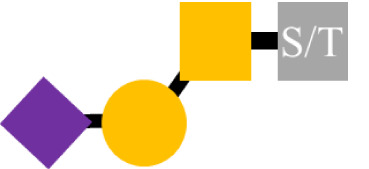	**Siglecs**	**iDC, MΦ, Mono, NK**
Le^X^	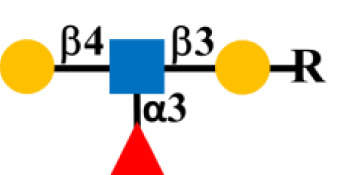	**DC-SIGN**	**iDC, MΦ**
SLe^X^	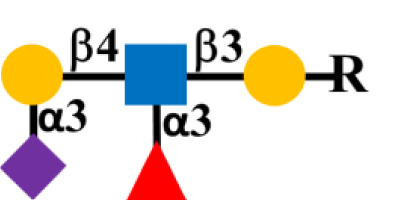	**DC-SIGN** **Siglecs**	**iDC, MΦ**
Le^Y^	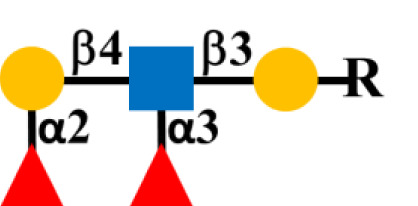	**DC-SIGN**	**iDC, MΦ**

### Tumor-Associated Carbohydrate Antigen-Associated Lectins

**Figure 7:**
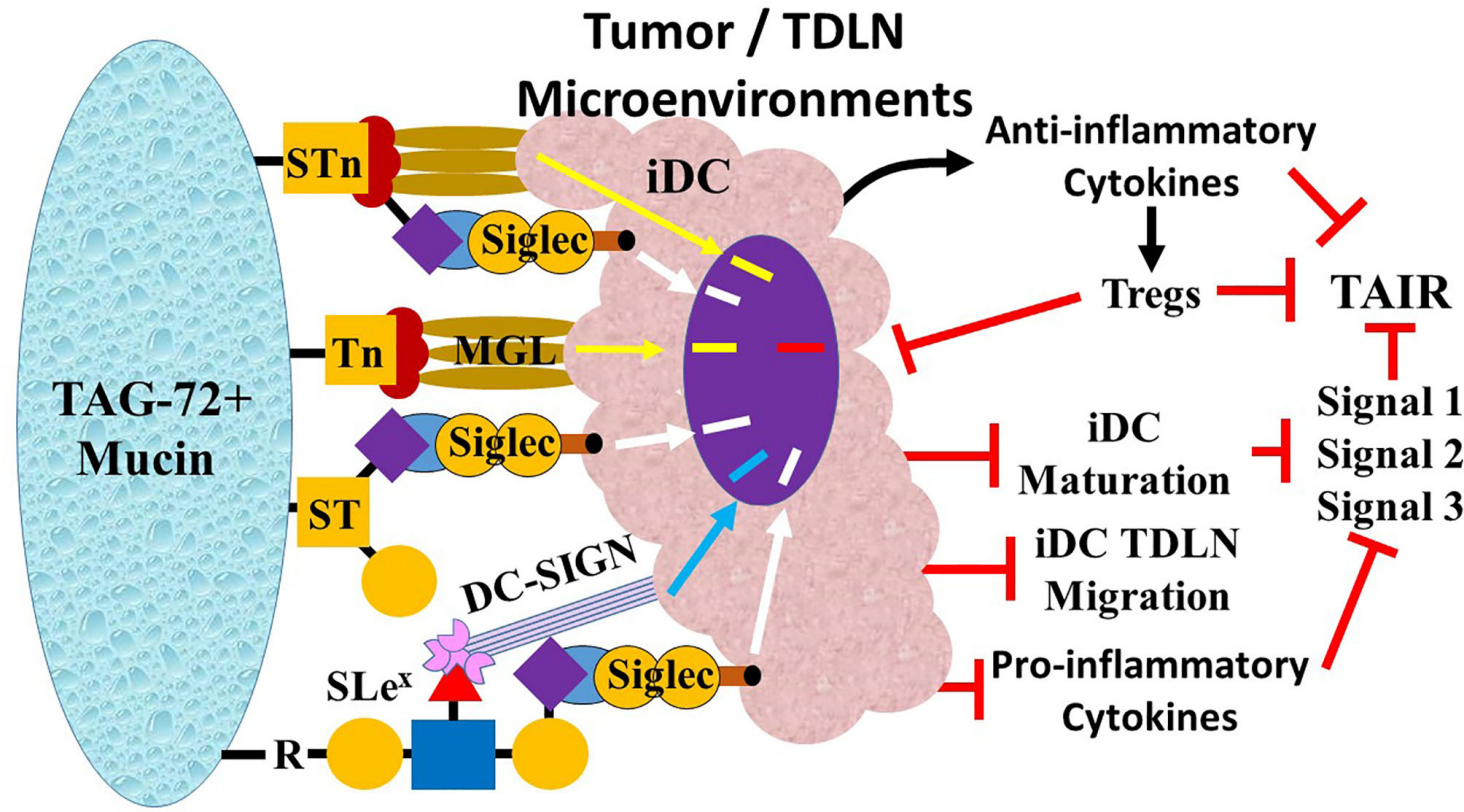
Overview of tumor-associated immune response (TAIR) suppression by Tn, STn, and ST. TAG-72-related suppression of the TAIR begins with the ligands Tn, STn, ST, and SLe^X^ on the TAG-72-positive mucin molecules binding to lectins on immature dendritic cells (iDCs). The sialic acid carbohydrate on ST, STn, and SLe^x^ bind to Sialic Acid Recognizing Ig-like Lectins (Siglec), while the galactose on Tn and STN binds to Macrophage Galactose-Type Lectin (MGL), and the fucose of the Lewis antigens (Lex, SLe^x^, and Le^y^) bind to Dendritic Cell-Specific ICAM-3 Grabbing Non-Integrin (DC-SIGN). The binding generates variable inhibitory signal pathways (MGL, yellow arrow; Siglecs, white arrow; DC-SIGN, blue arrow) that suppresses (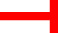) iDC maturation. Inhibition of iDC maturation inhibits iDC migration to tumor draining lymph nodes (TDLNs). Function changes including inhibition of the major histocompatibility complex (MHC) molecule expression (Signal 1), expression of co-stimulatory molecules (Signal 2), and secretion of pro-inflammatory cytokines (e.g., IL-6 and IL-12) (Signal 3) suppress the TAIR. Concurrently, there is increased release of anti-inflammatory cytokines (e.g., IL-10 and TGF-β) (black curved arrow) that further suppresses the T-helper 1 (Th1) TAIR and while activating (short black arrow) Th2, Th17, and regulatory T (Treg) cells. Treg activation suppresses the TAIR by further inhibiting iDC maturation (long black arrow) *via* cytotoxic T lymphocyte-associated protein 4 (CTLA-4) binding to CD80/86 co-stimulatory molecules and induces Th1 anergy by removing IL-2 from the environment. The result is suppression of the TAIR.

Lectins are carbohydrate-binding proteins that occur on the surface of every cell. Binding of PAMPs by the various types of lectins on leukocytes is critical to the mounting immune response to pathogens (89, 90). Similarly, lectins binding to DAMPs in the form TACAs are critical to mounting a TAIR. The critical lectins on leukocytes that bind TAG-72-associated TACAs in CRC include Sialic Acid Recognizing Ig-like Lectins (Siglecs), the C-type lectin Macrophage Galactose-type Lectin (MGL), and Dendritic Cell-Specific ICAM-3 Grabbing Non-Integrin (DC-SIGN). This binding on iDCs, NK cells, and macrophages leads to the suppression of the TAIR in CRC. The terminal sialic acids on various TACAs, including STn, ST, and mono- or di-sialylated Le^x/a^ and Le^y/b^ glycans, bind to the single carbohydrate recognition domain of Siglecs on DCs, macrophages, NK cells, and monocytes (91, 92). MGL, expressed on both DCs and macrophages, is specific for GalNAc on both Tn and STn (93, 94) (**Figure 7**). DC-SIGN lectins on DCs and macrophages bind high mannose and fucose carbohydrates of non-sialylated Lewis antigens Le^x/a^ and Le^y/b^ on carcinoembryonic antigen (CEA) and other glycoproteins (95–97).

The amount of sialylated glycans on the surface of normal cells far exceeds that of pathogens, and because of this, sialylated glycans are self rather than non-self glycans (SAMPs) that bind Siglecs (79). Leukocytes are the principal carriers of one or more of the 14 functional human Siglecs. Siglecs recognize terminal sialic acids on glycoproteins and glycolipids in a linkage-specific manner (88, 98–100). The single carbohydrate-receptor domain of Siglecs may bind a sialic acid on another glycan on the same cell (cis-binding) or to another cell or extracellular glycan (trans-binding) (92). Siglecs divide into two groups based on their amino acid sequence and chemical phylogeny—CD33-related or non-CD33-related (91). The CD33-related Siglecs predominate on cells of both the innate and adaptive immune responses, where they either activate or suppress the immune response to pathogens and self-antigens (99, 101). Inhibitory Siglecs-2, -3, and Siglecs-5 through 10 have a cytoplasmic tail that contains an immunoreceptor tyrosine-based inhibitory motif (ITIM). The cytoplasmic tail of Siglecs-14, -15, and -16 contains an immunoreceptor tyrosine-based activation motif (ITAM), while Siglecs-1 and -4 lack both (91).

The relative expression of Siglecs on circulating conventional dendritic cells (cDCs) (Siglecs-2, -3, -7, -9, and -15), plasmacytoid dendritic cells (pDCs) (Siglec-1 and Siglec-5), and macrophages (Siglecs-1, -3, -8, -9, -11, -15, and -16) in a steady state will change based on environmental triggers (92). NK cell subpopulations express Siglecs-7 and -9, and monocytes and their derived DCs and macrophages express Siglecs-1, -3, -7, -9, and -10 (60, 100). ITIM-carrying Siglecs suppress the TAIR, thus serving as a critical immune checkpoint (60, 92, 102) . Also, subsets of T cells express the inhibitory Siglecs-9 and -10 (103). In CRC, the terminal sialic acid residues on STn, ST, SLe^x/a^, and SLe^y/b^ TACA primarily bind to the single carbohydrate recognition domain of Siglecs-3, -7, and -9 on iDCs, macrophages, monocytes, and NK cells and by Siglec-15 on tumor-associated macrophages (TAMs).

MGL (CD301) is a C-type lectin that binds explicitly terminal α- and β-linked galactose and N-acetyl-galactosamine carbohydrates on both Tn and STn TACAs in CRC (104–106). MGL binding of a TACA leads to trimerization of the carbohydrate-binding domains followed by endocytosis of the bound ligand. Conformational changes arising after different binding ligands may activate different signal pathways and account for the ability of MGL to distinguish normal tissue from TACAs (105, 107–109). The expression of MGL is limited to iDCs and macrophages, where it modulates both the innate and adaptive immune responses, including the TAIR (110–112).

Like MGL, DC-SIGN (CD209) is a C-type lectin on subsets of DCs and macrophages (112, 113). DC-SIGN binds fucose (e.g., Lewis antigens) and mannose containing glycolipids and glycoproteins, as well as galactose and glucose to a much weaker extent. This “ligand-binding promiscuity” may arise from DC-SIGN’s tetrameric configuration with four independent acting carbohydrate-recognition domains (95). The presence of terminal sialic acid blocks this binding. DC-SIGN plays a critical role in viral infections, especially HIV and bacterial and fungal infections. DC-SIGN’s cell adhesion role arises from its ability to bind ICAM-2 on endothelial cells and ICAM-3 on naive T cells (112, 113).

### Escaping the Tumor-Associated Immune Response in Colorectal Carcinoma—A Cascade of Effects

CRC cells’ ability to escape the TAIR occurs in the microenvironments of both the tumor and the TDLNs (114, 115). The heterogeneous microenvironments in these locations contain many factors released by the tumor cells, stromal cells, and immune cells that suppress the TAIR and promote tumor cell proliferation and metastasis. CRC mucins and their associated TACAs play a critical role in this process by binding to and inhibiting the activity’s various innate immune cells that suppress the TAIR. Subsets of iDCs, along with subsets of NK cells, macrophages, and regulatory T cells (Tregs), play a central role in CRC’s elimination phase. We hypothesize that the various inhibitory signals arising from the binding of non-sialylated sugars on TACAs to MGL, DC-SIGN, and sialylated TACAs to Siglecs on subsets of these innate immune cells have an additive effect. In CRCs expressing TAG-72, this effect overcomes the opposing activating signals associated with other DAMPs and SAMPs binding to their respective lectins. TAG-72 in the blood and lymph nodes suggests that immunosuppression is concurrent in the microenvironments of the tumor and TDLNs (116).

### Inhibiting Immature Dendritic Cell Maturation

The binding of TACAs to their respective lectins on iDCs multiplies the impact of other tumor-derived cytokines and factors while inhibiting their maturation. In addition, the resulting inhibitory signal modifies activation signals resulting from binding DAMPs and SAMPs to other PRRs (95). iDC subsets express Siglecs-3, -7, -9, and -15, MGL, and DC-SIGN that bind the TACAs carried by the TAG-72 (92, 117). van Vliet et al. (111) demonstrated that the binding of GalNAc on Tn and STn by MGL on iDCs generated a signal that inhibits iDC maturation (111). In addition, MGL binding to Tn carried by CD45 on effector T cells suppresses their activities, further promotes the escape phase of the TAIR, and is associated with reduced OS in Stage III CRC (118, 119). DC-SIGN is primarily expressed on DCs in TDLNs (95, 120, 121) where it binds fucose on non-sialylated Lewis antigens carried by TAG-72 and other ligands such as carcinoembryonic antigen (CEA) (122). These inhibitory signals combine with Siglecs-3, -7, -9, and -15 binding to sialylated TACAs, and these binding events have a wide-ranging impact on the TAIR.

Inhibited iDC maturation leads to limited MHC I and MHC II expression, leading to the defective presentation of tumor antigens to naive CD8+ and CD4+ T cells. Concurrently, there is downregulation of CD80/86 costimulatory molecule expression and release of IL-12 and other cytokines needed for T-cell activation. The result is Th1 anergy for tumor antigens. An increase in the secretion of anti-inflammatory cytokines, including IL-10, TGF-β, and IL-4 promotes the differentiation of Tregs and the Th2 immune response that further suppresses the TAIR (123–125).

### Regulatory T Cells

Tregs are CD4+ CD25+ T cells that play a central role in suppressing the TAIR, and their accumulation in the microenvironments of the tumor and TDLNs portends a poor prognosis in CRC (126). Early studies of TAG-72-positive TDLNs of CRC demonstrated an increase in the CD4+:CD8+ ratio that may well be attributable to an increase in Tregs in these lymph nodes (53). Treg subsets exhibit overlapping mechanisms for suppressing the TAIR (127). Tregs express cytotoxic T lymphocyte-associated protein 4 (CTLA-4) that binds to CD80/86 on iDCs that further inhibits iDC maturation. Tregs’ high expression of the interleukin-2 receptor (IL-2R-CD25) consumes IL-2 in the TME and TDLN microenvironment needed for T-cell proliferation. The lack of IL-2 and suppression of the co-stimulation signal 2 further lead to Th1 anergy and an increase in the Th2 response. Tregs suppress the TAIR by release of perforin and granzyme that induces apoptosis of CD4 and CD8 effector T cells and by the release of adenosine triphosphate (ATP) (128).

### Tumor-Associated Carbohydrate Antigen Binding to Natural Killer Cells and Macrophages

In the case of NK cells, their concentration in the TME of CRCs appears to be minimal despite the presence of tumor-infiltrating T cells (129). DC-derived IL-12 secretion promotes NK cell cytotoxicity and the production of IFN-γ. Suppression of iDC maturation associated with TACA binding to cDC1 inhibits the production of IL-12 (130, 131). Subsets of NK cells express varying amounts of Siglecs-7 and -9 that bind mono- or di-sialylated Lewis antigens and STn and ST on mucins (132). TACA binding inhibits NK cell cytotoxicity and downregulates the release of the cytokines tumor necrosis factor-alpha (TNF-α), IFN-γ, and granulocyte-macrophage colony-stimulating factor (GM-CSF) (100, 132, 133).

TAMs are a prominent cellular component of the TME of CRCs. They predominantly arise from resident macrophages in the lamina propria of and from circulating monocytes (134). In general, TAMS are either pro-inflammatory (M1) or anti-inflammatory (M2). M1 and M2 TAMS differ in their phenotype and functions; however, individual cell analysis indicates the TAM subsets go beyond the dichotomous M1/M2 in the extent of phenotypic and functional subsets (135). Monocytes and macrophages express Siglecs-3, -7, -9, and -15, MGL, and DC-SIGN binding to TACAs on the TAG-72 positive glycoprotein (136, 137). The binding of TACAs to their lectins on M2 TAMs (73) leads to increased secretion of anti-inflammatory cytokines and chemokines that suppress the TAIR while supporting tumor angiogenesis, tumor cell proliferation, and metastasis (138). However, the clinical implications of the M1:M2 ratio in CRC appear unsettled, especially when looking at differences in location between the invasive front and sites deeper in the tumor (139).

### Resetting the Tumor-Associated Immune Response

Immunotherapy joins targeted therapy, surgery, chemotherapy, and radiation therapy as the main strategies for treating CRC. The goal of immunotherapy is resetting the TAIR, i.e., reversing immunoediting (140). Wculek et al. (77) proposed that modulating DC function be added to the list of immunotherapeutic strategies that include activating inert T cells with anti-PD-1, adoptive cellular therapy, CAR-T cell therapy, and vaccines. Each approach depends on functional DCs to present tumor antigens to naive T cells. However, clinical studies directed at TACAs in CRC indicate minimal response (141, 142). In addition, current targeted therapy and immunotherapy have the potential of severe side effects and the development of resistance that are not associated with curative intent surgical resection. In contrast, TAG-72 ADCS with curative intent removes the suppressed TAIR-involved tumor and TDLNs in primary and recurrent CRC. In our opinion, the ^125^I-labeled mMoAbs to TAG-72 do not themselves reset the TAIR in CRC patients. These mMoAbs serve as a preferential locator of exposed Tn, STn, and ST epitopes on the TAG-72 molecules that are bound to receptors on the cells of the innate and adaptive immune response.

In summary, an accurate assessment of the extent of CRC in a patient needs to be improved. Intravenous injection of a ^125^I-labeled mMoAb to TAG-72 binds to the antigenic epitope(s) on both tumor cell and extracellular mucin(s) in the TME. The intraoperative use of an HGDP provides the surgeon with a tool to detect tissue inside and outside the normal surgical field that is involved in the disease process. TAG-72-induced suppression of the tumor immune response rather than just the presence of tumor cells is the actual disease process needing correction. This correction is brought about by removal of all radioactive tissue to the point there is no detectable radioactivity at the end of surgery. This allows for the immunoediting tumor immune response to reset itself to the point of increasing patient survival beyond that associated with current staging protocols (**Figures 3**, **4**). In contrast, the inability of removing the radioactive tissue precludes resetting of the tumor immune response and leads to recurrent disease and/or decreased patient survival.

## Discussion

The last decade saw researchers define the mechanisms underlying the role of aberrant glycosylation in tumor invasion, metastasis, and evasion of the immune response. Publications describing the pattern of aberrant glycans—the tumor-associated glyco-code—in suppressing the antitumor immune response caused us to reexamine its possible role in explaining the results of our long-term survival data using TAG-72 ADCS for the treatment of patients with primary and recurrent CRC. Our survival results provide prospective clinical data supporting the relationship between the pattern of aberrant glycan expression and patient outcome. Also, our results highlight the importance of the sialoglycan–Siglec relationship in TAIR (143) and the concept of a “sialoglycan-Siglec glyco-immune checkpoint” (144) that TAG-72 ADCS inhibits. In addition, concurrent MGL bindings to GalNAc on Tn and STn leading to a Th2 TAIR further supports our hypothesis.

As with all hypotheses, there are caveats to consider. The first is that of the sample size of our clinical studies. Even though we have safely injected well over 500 patients with mMoAbs to TAG-72, we have limited follow-up data. The endpoints for our Phase I and Phase II clinical studies focused on the impact of changing surgeon behavior in real-time while increasing the accuracy in defining the extent of disease rather than OS altering the adjuvant therapy available before 2000. Also, there was a lack of knowledge to ask the right basic science questions at the time. Secondly, the data presented here are from only one institute. However, results from multiple institutions duplicated the stated endpoints of our study; however, there was no emphasis on obtaining patient survival data. Thirdly, there is no direct evidence, using human tissue samples, that our hypothesis is valid. In addition, our theory is based on results obtained from CRC and may not apply to other carcinomas. However, TAG-72 ADCS supports and extends the conclusion by Perdicchio et al. (145) “that reducing sialylation may provide a therapeutic option to render tumors permissive to immune attack.” This statement is true not only for CRC but also for similar carcinomas of gastrointestinal and genitourinary tracts, lungs, and breasts.

The most important caveat is that TAG-72 ADCS is not a “silver bullet.” However, our results clearly distinguish subpopulations of patients who vary in their prognosis. Reviewing the numbers, only 90% of primary colorectal adenocarcinomas express TAG-72, only 80% of these patients undergo surgery with curative intent, and mMoAb CC49 localizes in only 86% of these cases. The result is that approximately 62% of patients with primary CRC have a significant chance of improved OS. However, on an annual basis, that equates to over 1 million patients per year worldwide and over 95,000 patients in the US who could benefit from TAG-72 ADCS.

Testing our hypothesis could involve repeating our clinical studies using one of the humanized fragments of the mMoAb CC49 developed in the last decade (14, 146–148). There are many radionuclides that can be used instead of ^125^I, but it is critical to match the antibody’s clearance to that of the radionuclide. Preoperative imaging techniques provide only information in the X-Y plane and often miss small lesion. The use of a ^125^I-labeled mMoAb provides in-depth information that allowed us to detect and then locate tumor-involved tissue within the liver and in extraregional lymph nodes draining the tumor. Intraoperative imaging combined with the HGDP further improves real-time tumor detection and assessment of surgical resection to ensure the best outcome for the individual patient (149). In addition to using an HGDP, intraoperative imaging confirms adequate removal of the TAG-72 tissue and for accurate sampling of the tissue in the Pathology laboratory (149). This type of study precludes current robotic and laparoscopic approaches and requires a thorough assessment using a gamma detection probe (150). It does not preclude the use of fluorescence-labeled intact or fragments of monoclonal antibodies nor does it preclude the use of bilabeled antibodies (151). Fluorescence-guided surgery is limited by the ability of the fluorophore’s light to penetrate tissue, which is not a problem for a radionuclide. However, fluorophores allow for more sensitive assessment of surfaces (e.g., serosal, tissue margins). These combined techniques can be used to provide the researcher with the “correct” tissue for subsequent study possibly by multicolor fluorescent molecular digital imaging of frozen sections. The resulting tissue can also be used to establish patient-derived xenografts (PDXs) in an immunodeficient mouse model (reviewed in (152) that is best suited to assess impact role of TAG-72 in suppressing the TAIR.

Our overall goal is to provide clinicians with tumor-specific information beyond the current staging system to more accurately stratify CRC patients at the molecular level and specifically allow for a more case-by-case determination of the most appropriate cancer-targeted therapies. Such a further refinement in this concept of oncologic theranostics, as it relates to TAG-72, other aberrant glycans, and the resultant TAIR, may well include new innovative and personalized immunotherapies directed at specific glycan targets expressed within individual tumors. This innovative oncologic theranostics would hold significant promise for improving CRC patients’ care and long-term outcomes (17). Our results suggest that TAG-72 ADCS will provide the opportunity to obtain appropriate tissues needed for an accurate definition of the roles of aberrant glycans, their lectins, and the specific innate immune cells that express them on the biology of tumor cells. The goal is to develop new molecules and methods to inhibit the immunosuppression associated with aberrant glycosylation.

Our results and those of the authors who wrote the papers referenced here provide proof of the sageness of Dr. Robert M. Zollinger, a giant of American Surgery, who stimulated his younger colleagues with, to paraphrase, “The answer is right in front of you, but you don’t know enough to see it.” We are still learning.

## Data Availability Statement

The raw data supporting the conclusions of this article will be made available by the authors without undue reservation.

## Ethics Statement

The studies involving human participants were reviewed and approved by the institutional review board. The patients/participants provided their written informed consent to participate in this study.

## Author Contributions

CH, SP, CM, and EM conceptualized the article. CH wrote the article, and each author edited the article. CM and EM conducted the clinical studies. All authors contributed to the article and approved the submitted version.

## Funding

Funding for the prior clinical studies presented in this current article was previously provided by grants from the NIH and Neoprobe Corporation (Navidea Biopharmaceuticals, Inc., as of January 2012). The funder was not involved in the study design, collection, analysis, interpretation of data, the writing of this article, or the decision to submit it for publication.

## Conflict of Interest

Authors CH, EM, and CM are unpaid employees of Actis Medical, LLC, a small medical device company. Author CM was employed by the company Neoprobe Corporation for a portion of the clinical studies.

The remaining authors declare that the research was conducted in the absence of any commercial or financial relationships that could be construed as a potential conflict of interest.

## Publisher’s Note

All claims expressed in this article are solely those of the authors and do not necessarily represent those of their affiliated organizations, or those of the publisher, the editors and the reviewers. Any product that may be evaluated in this article, or claim that may be made by its manufacturer, is not guaranteed or endorsed by the publisher.

## Abbreviations

ADCS, antigen-directed cancer surgery; ARGs, autoradiography grains; CRC, colorectal carcinoma; DAMPs, damage-associated molecular patterns; DC, dendritic cell; cDC, conventional dendritic cell; iDC, immature dendritic cell; mDC, mature dendritic cell; pDC, plasmacytoid dendritic cell; DC-SIGN, dendritic cell-specific ICAM-3 grabbing non-integrin; H&E, hematoxylin and eosin; HGDP, handheld gamma detecting probe; IHC, immunohistochemical; IL, interleukin; MGL, macrophage galactose-type lectin; mMoAb, murine monoclonal antibody; MHC, major histocompatibility complex; MUC, mucin; NK, natural killer; OS, overall survival; PAMPs, pathogen-associated molecular patterns; pStage, pathologic stage; RIGS, radioimmunoguided surgery; SAMPs, self-associated molecular patterns; TACA, tumor-associated carbohydrate antigen; TAG-72, tumor-associated glycoprotein-72; TAIR, tumor-associated immune response; Th, T-helper cell; TME, tumor microenvironment; Tn, GalNAcα1-O-Ser/Thr or Thomsen-nouveau; STn, Sialylated Tn; Treg, regulatory T cell; Siglecs, sialic acid recognizing Ig-like lectins.
